# Innovative surgical and stress-stimulated rat model of ligamentum flavum hypertrophy

**DOI:** 10.3389/fvets.2024.1490769

**Published:** 2025-01-16

**Authors:** Long Chen, Zhaoyuan Zhang, Niandong Li, Wanxia Zhang, Zhouhang Zheng, Yu Zhang

**Affiliations:** ^1^Guangzhou University of Chinese Medicine, Guangzhou, China; ^2^Guangdong Provincial Second Hospital of Traditional Chinese Medicine, Guangzhou, China

**Keywords:** hypertrophy of ligamentum flavum, rat model, western blot, MRI, immunohistochemistry

## Abstract

**Background and purpose:**

Animal models of LFH are still in the exploratory stage. This study aimed to establish a reliable, efficient, and economical model of LFH in rats for the study of human ligamentum flavum (LF) pathological mechanisms, drug screening, development, improvement of surgical treatment, disease prevention, and other aspects.

**Methods and materials:**

Forty rats were divided into an experimental group and a sham group of 20 rats. The experimental group (*n* = 20) was treated with an innovative operation combined with stress stimulation at the L5-L6 segments, the L5 and L6 spinous processes, transverse processes, and supraspinous ligaments were excised, along with removal of the paraspinal muscles at the L5-L6 level. One week after surgery, the rats were subjected to slow treadmill running daily. In the experimental group (*n* = 20), the spinous process, transverse process, supraspinous ligament and paraspinous muscle of L5 and L6 were excised. And for a week after the surgery, the rats ran on a treadmill at a slow pace every day. While the sham group (*n* = 20) was treated with sham operation only. Seven weeks later, MRI, immunohistochemistry (IHC), and western blot (WB) will be performed on the LF of the L5-6 segment in the two groups of rats.

**Results:**

MRI results showed that the LF in the experimental group was significantly thicker than that in the sham group. Masson staining results indicated that LF thickness, collagen fiber area, and collagen volume fraction (CVF) were significantly higher in the experimental group than in the sham group. IHC and WB showed that the expression of TGF-β1, COL1, and IL-1β in the LF of the experimental group was significantly higher than that in the LF of sham group.

**Conclusion:**

Through innovative surgical intervention combined with stress stimulation, a relatively reliable, efficient, and convenient rat LFH model was established.

## Introduction

Lumbar spinal stenosis (LSS) is common in individuals over 40 years of age and has a high incidence rate worldwide (1). Studies have shown that the prevalence of LSS among the general population in the United States is approximately 11% (2). LSS is caused by narrowing of the central vertebral canal, intervertebral foramina, and neural foramina due to hypertrophy of ligamentum flavum (LFH), degenerative disc protrusion, bone spurs, facet joint hypertrophy, ligament calcification, and other factors, leading to nerve root and cauda equina compression and resulting in neurological dysfunction (3, 4). LFH is the primary pathological cause of LSS, Chronic degeneration, inflammation, metabolic disorders, and other factors are the important causes of LFH (5, 6). The important histological manifestation of LFH is an increase in collagen fibers and loss of elastic fibers, especially with increased COL1 expression (7, 8). In terms of molecular mechanisms, the high expression of inflammatory factors such as IL-1*β* and the growth factor TGF-β is also considered an important feature of LFH (9).

At present, an animal model of LFH is still in the exploratory stage. A reliable animal model of LFH is crucial to studying disease mechanisms, drug screening and development, and disease prevention. Reliable animal models must possess advantages such as similarity, repeatability, operability, and standardization. Currently, mice, rats, and rabbits are the main animals used to construct lumbar LFH (10–12). Currently, there are two main types of animal models of LFH: surgical and non-surgical. Surgical modeling involves surgical disruption of the original mechanical structures of the animal’s lumbar spine, resulting in mechanical stress concentration or lumbar spine instability, leading to LFH. Hayashi et al. used rabbit posterolateral spinal fusion surgery to concentrate mechanical stress on the L3-4 segments for modeling (13). Zhang et al. (14) created an LFH rat model by excising rat paraspinal muscles. Sato et al. (15) induced LFH in rats by excising the spinous processes, supraspinous ligaments, and interspinous ligaments. Wang et al. (16) induced LFH by destabilizing the L5-6 segments of rats through excision of the paraspinal muscles, spinous processes, and grinding of bilateral articular processes. Non-surgical models typically use the bipedal standing model to simulate the effects of long-term spinal loading on the LF. This model is simple, avoids repeated anesthesia, but posture and individual differences may affect the reproducibility of the results. Saito et al. (10) established an LFH mouse model by simulating stress conditions and fully loading the mechanical stress on the lumbar LF.

Although surgical modeling creates conditions of lumbar spine instability, it may be limited by activity patterns and levels of activity in rats, potentially reducing the success rate of experiments. In this study, we innovatively combined surgical techniques with stress stimulation to develop a reliable, efficient, and convenient rat model for LFH. Simultaneously, by comparing the similarities and differences in the relevant IHC, WB, and MRI results with those in humans, the feasibility of this approach was validated. The following is a report of this experiment:

## Methods and materials

### Animals and grouping

Forty male Sprague–Dawley (SD) rats, aged 8 weeks and weighing approximately 250 g, were randomly assigned to two groups: an experimental group (*n* = 20) and a sham group (*n* = 20). The rats were housed in a controlled environment with regulated temperature and humidity. All procedures were performed in accordance with the ARRIVE guidelines.

### Intervention procedure

**Figure 1:**
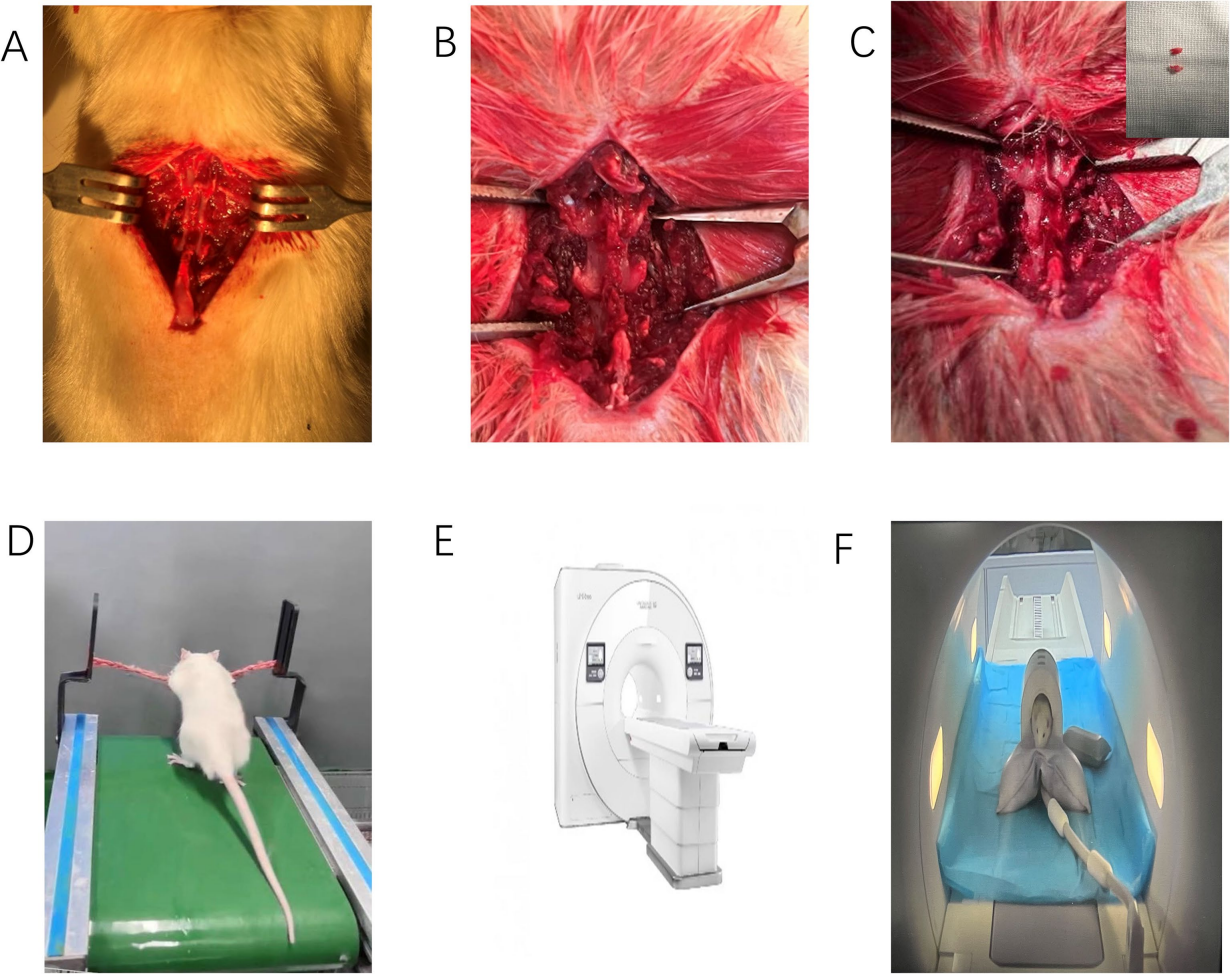
Surgical procedure and MRI equipment. **(A–C)** Illustrate the surgical procedure for the experimental group (*n* = 20), including the excision of the L5-L6 spinous processes, transverse processes, and supraspinous ligaments. **(D)** Shows the standing treadmill running of rats post-surgery. **(E,F)** Depict the MRI equipment used to measure ligamentum flavum thickness.

Surgical procedures were performed under isoflurane inhalation anesthesia. Anesthesia was induced with 3–4% isoflurane in an induction chamber and maintained at 1.5–2% isoflurane with a flow rate of 0.3 L/min using a nose cone. All surgeries were performed under sterile conditions. In the experimental group (*n* = 20), the L5 and L6 spinous processes, transverse processes, and supraspinous ligaments were excised, along with the removal of the paraspinal muscles at the L5-L6 level, and the superior and inferior articular processes were excised, resulting in disruption of the facet joints to induce lumbar instability and enhance stress stimulation on the lumbar spine (Figures 1A–C). One week after surgery, the rats were subjected to slow treadmill running daily (Figure 1D). Adaptive training for 3 days, the first day: 5–6 m/min, 10 min; the second day, 6-7 m/min, 20 min; the third day, 8 m/min, 25 min; day 4 starts, 8 m/min, 30 min. The sham group (*n* = 20) received surgical exposure only, with no additional treadmill exercise or stress stimulation. This design allowed us to isolate the effects of surgical intervention combined with stress stimulation from those in the sham group. Postoperatively, penicillin (20,000 U/rat, Beijing Yuekang Pharmaceutical Co., Ltd., 0.48 g/800,000 U) was injected to prevent infection, and the surgical site was disinfected daily with iodophor until the wound healed. All SD rats were humanely euthanized 7 weeks after surgery, following ethical guidelines for animal research.

### MRI analysis

Seven weeks after surgery, the rats were anesthetized with isoflurane and underwent MRI scans. Anesthesia was induced with 3–4% isoflurane in the induction chamber and then maintained with 1.5–2% isoflurane with a nose cone during imaging. Following anesthesia, MRI scans were performed using an MRI system (uMR790 3.0 T, Shanghai United Imaging Healthcare Co., Ltd.) equipped with a dedicated rat coil to measure the L5-6 LF thickness. The MRI parameters were set as follows: TR: 4417.0 ms, TE: 118.16 ms, FA: 150°, WW: 475, WL: 215, BW: 130 Hz, FOV: 35×35 mm.

### Histological analysis

For histological analysis, the L5-L6 lumbar vertebrae were extracted from the rats, fixed in 10% formalin, decalcified, and then embedded in paraffin. Sections of 4 μm thickness were prepared for staining. Initially, Hematoxylin and Eosin (HE) staining was used for general tissue localization, followed by Masson’s trichrome staining to visualize collagen fibers in the LF. In Masson’s staining, collagen fibers appeared blue, while elastic fibers appeared red.

The LF thickness, collagen area and elastic fiber area on both sides were measured by imageJ1.53 and then averaged. The LF thickness was measured by drawing a line parallel to the inner border of the LF and then drawing a perpendicular line at the thickest point of the LF. The distance between the inner and outer boundaries of the LF, as indicated by the perpendicular line, was used to calculate LF thickness. ImageJ analysis was performed on both sides of the LF for each rat, and the average value was taken to represent the data for each rat. Additionally, the collagen volume fraction (CVF) was calculated by measuring the area of collagen fibers relative to the total area of the LF. The data from both sides of the LF were averaged to obtain a representative value for each rat.

Immunohistochemistry (IHC) experiments were conducted by incubating the sections with specific primary antibodies, followed by incubation with secondary antibodies conjugated to enzymes or fluorescent substances. For enzyme-labeled secondary antibodies, colorimetric staining was performed using suitable substrates, while fluorescent-labeled secondary antibodies were observed directly under a fluorescence microscope.

After staining, the sections were sealed with a mounting medium and analyzed under a microscope. ImageJ 1.53 software was used for quantitative analysis of the staining intensity, with the mean optical density (MOD) serving as the evaluation parameter for positive expression. Three random fields were captured at 600x magnification, and the average of these fields was considered the final result.

### Experimental equipment and reagents

For the histological analysis, various equipment and reagents were used. The tissue samples were processed using a dehydrator (Wuhan Junjie, JJ-12 J) and embedded with an embedding machine (Wuhan Junjie, JB-P5). Tissue sections were prepared using a pathological slicing machine (Shanghai Leica, RM2016). Dehydration and clearing were performed with anhydrous ethanol and xylene from China National Pharmaceutical Group. Antigen retrieval was done using several solutions, including EDTA (pH 8.0) (Cat. No. B2001), EDTA (pH 9.0) (Cat. No. B2002), and Citrate (pH 6.0) (Cat. No. B2010), all obtained from Wuhan Baiqiandu. For washing the tissue sections, PBS (Cat. No. B0002) from Wuhan Baiqiandu was used.

For the immunohistochemical experiments, the following primary antibodies were used: COL1A1: Rabbit anti-COL1A1 antibody (1:600 dilution), Catalog No. GB11022-3, Google Inc., Rabbit, with antigen retrieval in Citrate pH 6.0 at medium heat for 5 min; TGFβ1: Rabbit anti-TGFβ1 antibody (1:100 dilution), Catalog No. BA0290, Bioss, Rabbit, with antigen retrieval in Citrate pH 6.0 at medium heat for 5 min; IL-1β: Rabbit anti-IL-1β antibody (1:200 dilution), Catalog No. GB11113, Google Inc., Rabbit, with antigen retrieval in Citrate pH 6.0 at medium heat for 5 min.

Secondary antibodies were used for detection following primary antibody incubation. These included Anti-rabbit IgG: Goat anti-rabbit IgG (H + L), HRP conjugated (1:100 dilution), Catalog No. 5220–0362, SeraCare, USA; Anti-rabbit IgG: Goat anti-rabbit IgG (H + L), HRP conjugated (1:500 dilution), Catalog No. 5220–0336, SeraCare, USA; Anti-mouse IgG: Goat anti-mouse IgG (H + L), HRP conjugated (1:500 dilution), Catalog No. 5220–0341, SeraCare, USA; and a Universal Secondary Antibody: Goat anti-rabbit/mouse IgG (H + L), HRP conjugated (1:1 dilution), Catalog No. K5007, DAKO, Denmark.

### Western blot (WB) experimental procedure

Protein samples were extracted from two groups of specimens, and three repeated WB experiments were performed for the majority of target proteins. However, due to tissue availability, only one biological replicate per group was used for the analysis of Vimentin and *α*-SMA. Proteins were separated using sodium dodecyl sulfate-polyacrylamide gel electrophoresis (SDS-PAGE) and transferred onto a PVDF membrane. To prevent nonspecific binding, the membrane was blocked with 5% non-fat dry milk in 1 × TBS-T (Tris-buffered saline with 0.1% Tween-20) at room temperature for 1 h. After blocking, the membrane was incubated with primary antibodies specific to the target proteins (including GAPDH, COL1, Vimentin, *α*-SMA, COL1A1, TGFβ1, and IL1β) overnight at 4°C. After washing, the membrane was incubated with enzyme-conjugated secondary antibodies for 1 h at room temperature. After further washing, chemiluminescent substrates were applied, and signals were detected either by exposure in a darkroom or using a chemiluminescence imaging system (CLI Instrument).

### Main instruments

The main instruments used for Western Blot (WB) included the Electrophoresis Apparatus (EA) for protein separation, the Transfer Electrophoresis Tank (TET) for protein transfer, and the Vertical Electrophoresis (VE) system. Protein visualization was done using a Chemiluminescence Imaging Instrument (CLI Instrument). Additional equipment included a High-Speed Freezing Microcentrifuge (HSF Micro) for sample preparation, a Thermo Fisher Scientific Shaker (TF Shaker), and an Adjustable Mixer (AM) for antibody incubation.

### Primary reagents

Primary reagents included GAPDH mAb for the loading control, COL1 Polyclonal Ab, Vimentin Polyclonal Ab, SMA Polyclonal Ab, COL1A1 Rabbit mAb, TGFβ1 Ab, and IL1β Ab for protein detection. Secondary antibodies (GAM IgG and GAR IgG) were conjugated with HRP. Other reagents included RIPA Buffer, BCA Kit, SDS-PAGE Loading Buffer, Dual-Color Marker, and PVDF Membranes for protein transfer and analysis.

## Statistics

Statistical analyses were conducted using SPSS 22.0 (IBM Corp., Armonk, NY, USA). The student’s *t*-test and Mann–Whitney U test were used to compare MRI, IHC, and WB data between the two groups, depending on the distribution of the data. Statistical significance was set at (*p* < 0.05). Statistical graphs were generated using GraphPad Prism 9.5.0 (GraphPad Software, San Diego, CA, USA).

## Ethics and registration

This study was approved by the Ethics Committee of the Second Affiliated Hospital of Guangdong Provincial Hospital of Chinese Medicine (Approval Number: 049044).

## Results

### MRI results

**Figure 2:**
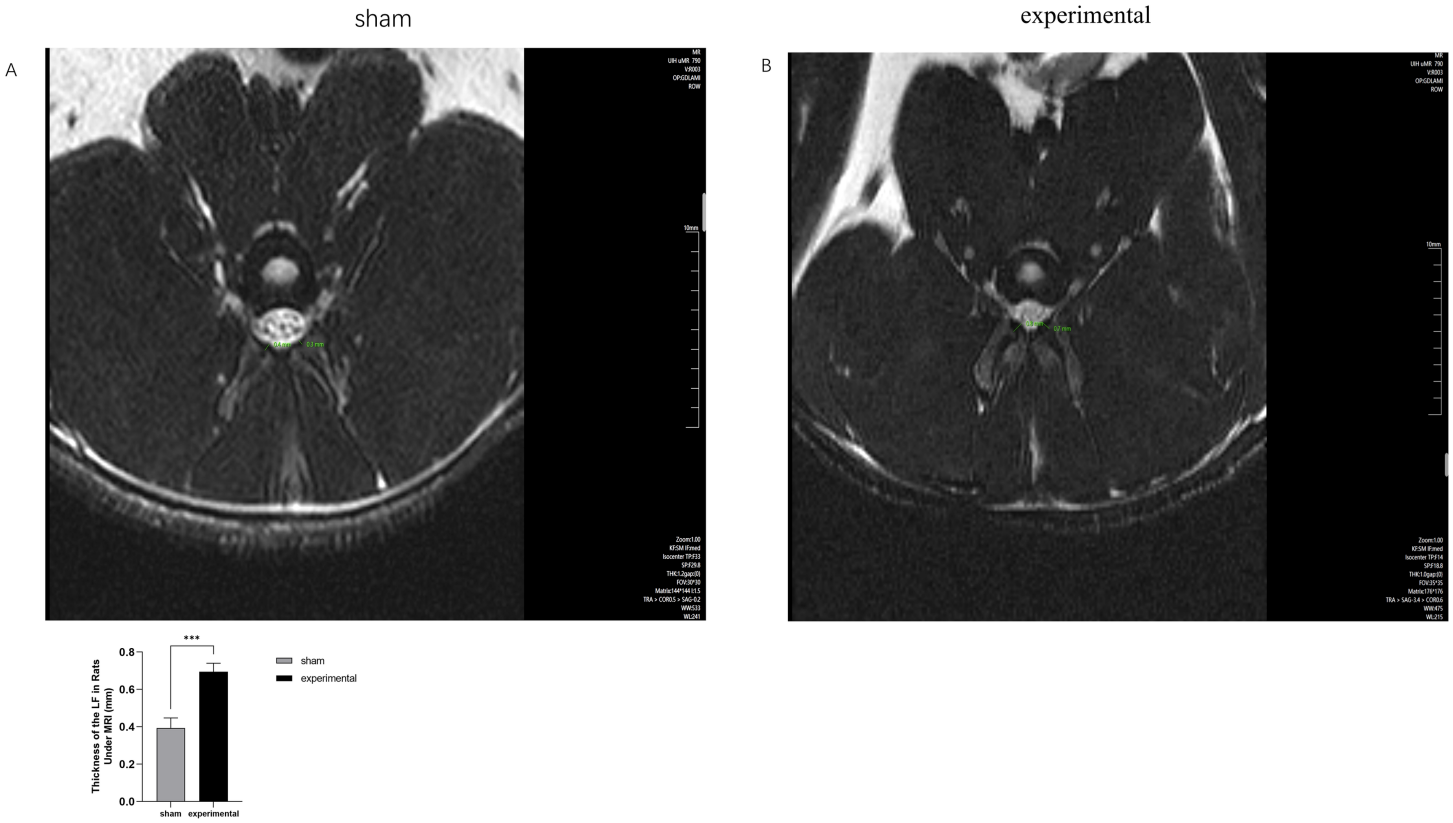
MRI of LF. **(A,B)** MRI comparison showing thickening of the LF and compression of the spinal canal in the experimental group. *n* = 10, *p* < 0.001.

Figures 2A,B shows MRI images of the ligamentum flavum (LF) in both the experimental and sham groups. The LF in the experimental group was significantly thicker than in the sham group (*p* < 0.001), and the thickened LF was observed to compress the spinal canal. These findings suggest the presence of ligamentum flavum hypertrophy in the experimental group.

### Masson’s trichrome staining results

**Figure 3:**
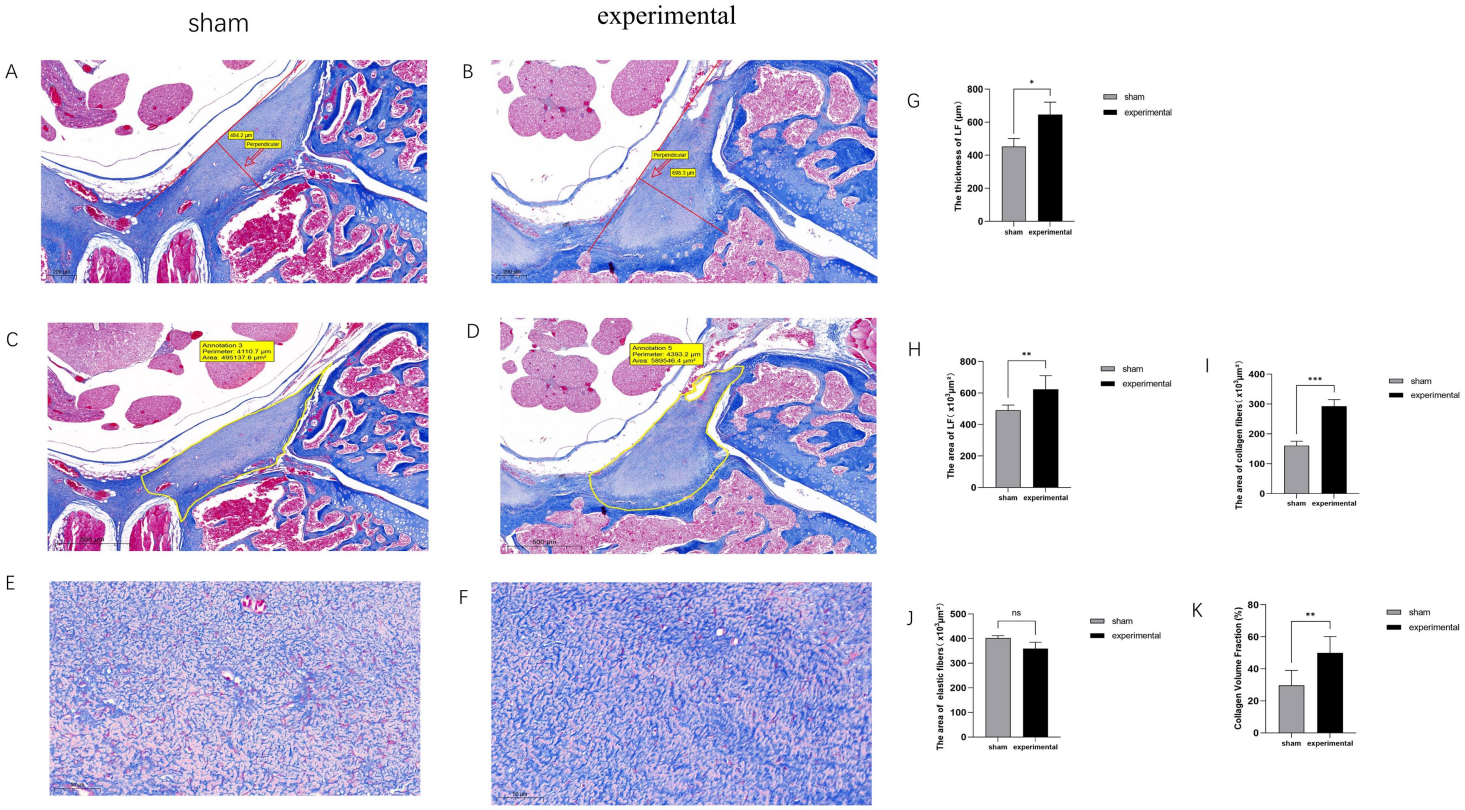
Masson trichrome staining. **(A–K)** Masson trichrome staining showing increased collagen fibers in the experimental group, with higher collagen area and CVF compared to the sham group, *n* = 3, *p* < 0.05, 0.01, or 0.001.

Figures 3A–K show Masson’s trichrome staining of the LF in both groups. The thickness and area of the LF in the experimental group were significantly greater than those in the sham group (*p* < 0.05 or 0.01). Moreover, the collagen fiber area in the LF of the experimental group was significantly larger than that in the sham group (*p* < 0.001), indicating increased collagen deposition. However, there was no significant difference in the area of elastic fibers between the two groups (*p* > 0.05). Additionally, CVF in the experimental group was significantly elevated (*p* < 0.01).

### IHC results

**Figure 4:**
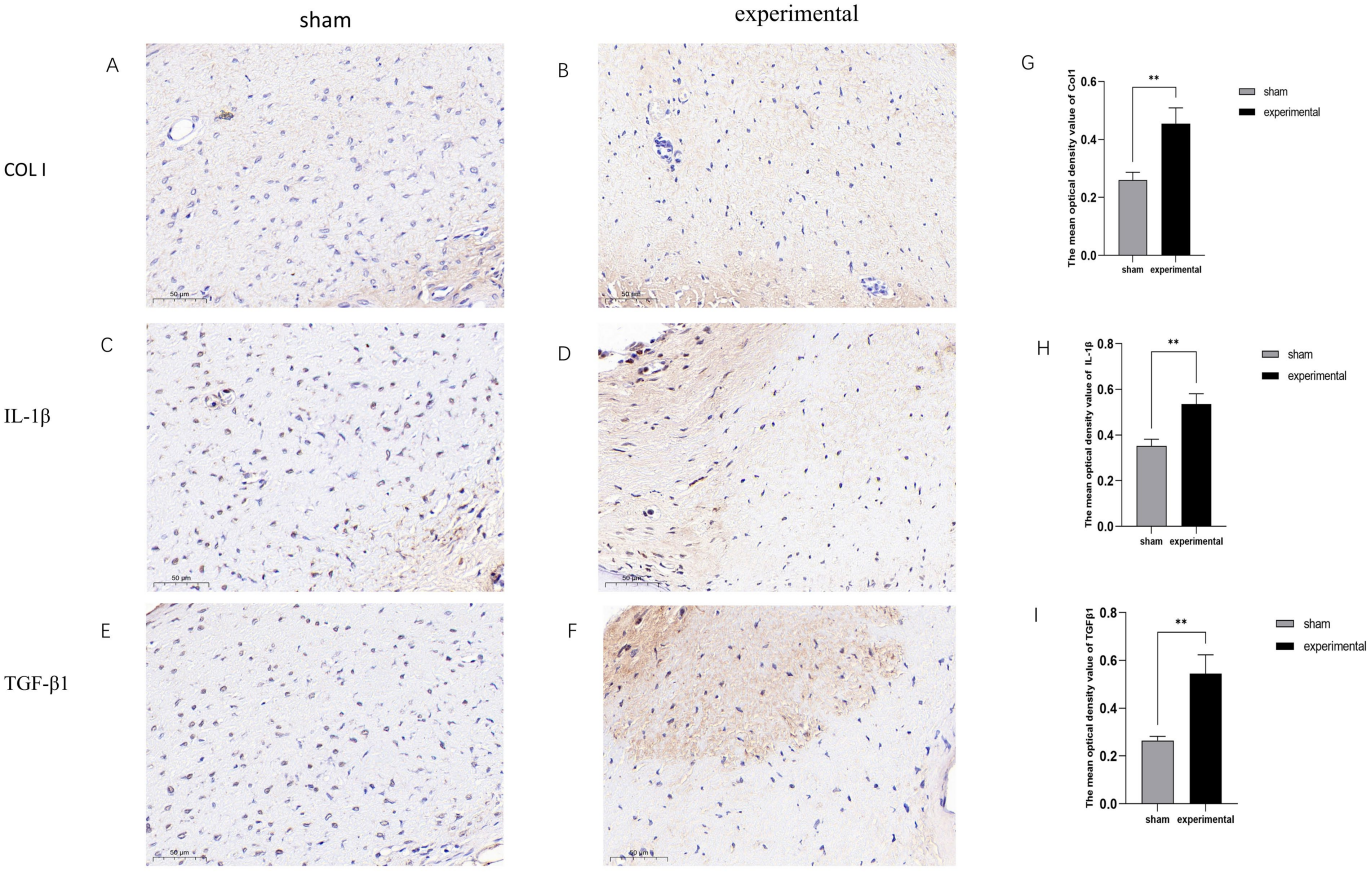
IHC results. **(A–F)** Positive IHC staining for TGF-β1, COL I, and IL-1β in the experimental group. **(G–I)** Statistical analysis showing significant differences, *n* = 3, *p* < 0.01.

Figures 4A–F display typical positive IHC results for Collagen I (COL I), TGF-β1, and IL-1β. The expression levels of these proteins were higher in the experimental group compared to the sham group. Figures 4G–I show statistical analysis demonstrating that the expression of TGF-β1, COL I, and IL-1β was significantly increased in the experimental group (*p* < 0.01).

### WB results

**Figure 5:**
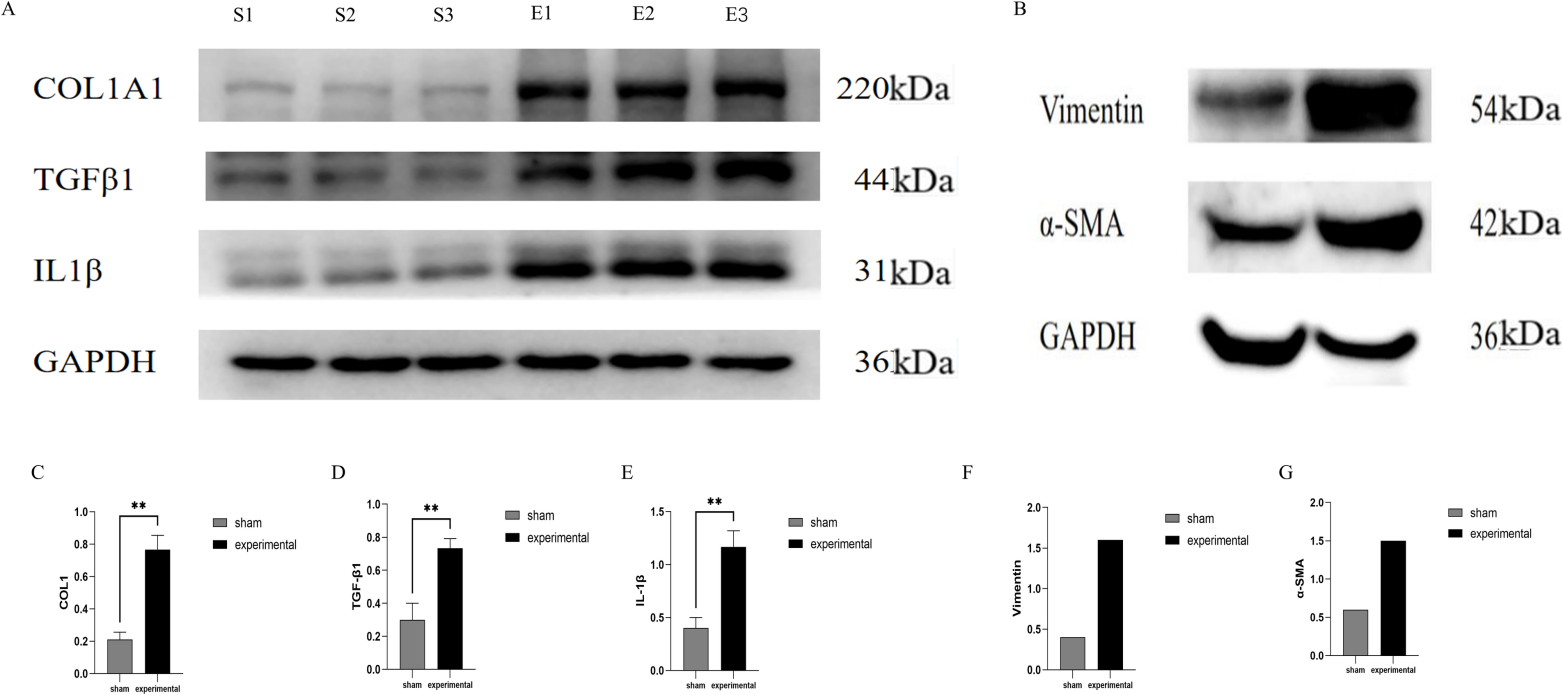
Western blot analysis. **(A)** Western blot showing higher expression of COL I, TGF-β1, IL-1β. **(B)** Vimentin, and α-SMA in the experimental group. **(C–E)** Quantification of COL I, TGF-β1, and IL-1β (*p* < 0.01), COL I, TGF-β1, IL-1β, *n* = 3. **(F,G)** Quantification of Vimentin and α-SMA, *n* = 1.

Western blot analysis was performed to assess the expression of COL I, TGF-β1, IL-1β, Vimentin, and *α*-SMA in the LF. The COL I, TGF-β1, and IL-1β bands were significantly more intense in the experimental group compared to the sham group, indicating upregulation of these proteins involved in fibrotic remodeling and inflammatory responses (Figure 5). Statistical analysis of COL I, TGF-β1, and IL-1β expression revealed significant differences (*p* < 0.01) between the experimental and sham groups, with higher levels in the experimental group (Figures 5A,C–E).

In addition to these markers, Vimentin and *α*-SMA were also evaluated as markers of fibrotic remodeling. The Vimentin band appeared at approximately 57 kDa, and α-SMA at 42 kDa (Figures 5B,F,G). Higher expression was observed in the experimental group, suggesting a role in fibrotic changes. However, due to the limited sample size (one sample per group for Vimentin and α-SMA), these results should be considered preliminary. Further studies with a larger sample size are needed to confirm these findings.

## Discussion

LFH is a significant contributor to the LSS. Mechanical stress, chronic inflammation, age, and other factors are known to cause LFH. The construction of an animal model of LFH is still in the exploratory stage, and there is currently no universally recognized modeling method. Currently, there are several methods for modeling LFH, including surgical modeling, non-surgical stress stimulation modeling, and LPA induction (LPA induction refers to the use of lysophosphatidic acid to stimulate LFH). Surgical modeling includes lumbar fusion and instability. For example, Hayashi et al. (13) used posterolateral bone graft fusion surgery in rabbits, concentrated mechanical stress on the L3-4 segments, and observed LFH 16 weeks postoperatively. Lumbar instability can also induce LF hypertrophy. Wang et al. (16) by resecting the paraspinal muscles, spinous processes, and bilateral articular processes of the L5-L6 segments in rats, induced instability in the L5-6 segments, and observed an increase in the thickness of the LF in rats 8 weeks after surgery. Non-surgical modeling primarily involves increased stress stimulation of the spine. Saito et al. (10) simulated stress stimulation conditions, loaded mechanical stress onto the lumbar LF of mice and established an LFH mouse model after 12 weeks of stimulation.

This study demonstrated that enlarging the surgical scope combined with treadmill running can establish a rat model of LFH, further reducing modeling time and improving success rate. LFH induction is expected to occur within seven weeks. By excising the paraspinal muscles, spinous processes, bilateral articular processes, and supraspinous ligaments, mechanical stress is concentrated in the L5-L6 segments of the lumbar spine. Concurrently, treadmill running increased the load on the segment and generated shear force during spinal rotation. This experimental model further intensified the lumbar spine’s stress stimulation, thereby enhancing the likelihood of success.

MRI plays a crucial role in LFH diagnosis. Increased LF thickness, spinal canal narrowing, and anterior spinal cord compression are typical manifestations of LSS in humans (17, 18). MRI provides high-resolution images, depicting the detailed structures of the LF, allowing for accurate assessment of its morphology, thickness, and possible injuries. In this study, MRI was used to evaluate the animal model from an imaging perspective, enabling a comprehensive evaluation of the LF from multiple angles and facilitating easier observation of the degree of spinal canal compression. MRI results showed significant thickening of the ligamentum flavum in the experimental group, which led to clear compression of the spinal canal. This compression is a key feature of LSS in humans, suggesting that the mechanical stress in our animal model could contribute to spinal canal narrowing. These imaging results enhanced the persuasiveness of the experimental findings and demonstrated the reliability of this model in mimicking the pathological features of human LFH, including the associated spinal canal compression.

Studies have shown that human LF consists of approximately 20–25% collagen fibers and 75–80% elastic fibers (8, 19). In this experiment, the proportion of collagen fibers to elastic fibers in Masson staining of the sham group was similar to that in humans, indicating a resemblance in the structure of rat LF to that of humans. Therefore, it is reasonable to use a rat model to study the pathological mechanisms of LFH. LFH is characterized by an increase in collagen fibers, loss of elastic fibers, and increased thickness and cross-sectional area of the LF (8, 20, 21). Our experiment yielded similar results, indicating the successful establishment of an LFH rat model using this experimental protocol.

The increase in collagen fibers, particularly the upregulation of COL 1 expression, is a significant manifestation of human LFH (22). Inflammatory factors, such as IL-1*β* and TGF-β, play a crucial role in the fibrotic process of LFH (23–25). Our results are consistent with these findings, indicating that this model effectively mimics the fibrotic pathological process of LFH. Although immune cell populations were not specifically quantified in this study, it would be valuable for future research to investigate whether immune cells, such as macrophages or T lymphocytes, accumulate in the LF tissue, as these cells contribute to chronic inflammation and fibrosis. The increased expression of IL-1*β* and TGF-β in this study is likely due to activation of local immune cells like macrophages or fibroblasts in response to mechanical stress. While systemic inflammation was not assessed, further studies should explore whether circulating cytokines or immune cells also contribute to LFH progression.

In addition to these markers, Vimentin and *α*-SMA, which are key indicators of fibrotic remodeling, were also analyzed. Although higher expression of these proteins was observed in the experimental group, due to the limited sample size (one sample per group), these results should be considered preliminary. Further studies with a larger sample size are necessary to validate these findings and fully assess their role in the pathological process of LFH.

Previous rodent models of LFH mostly relied on surgical interventions to induce lumbar spinal instability. However, in the process of constructing animal models, we found that postoperative rats may not have sufficient activity time and space in their housing cages to generate adequate stress on their lumbar spine. Therefore, in this experiment, we combined rat standing running with surgical intervention to increase the stress on the lumbar spine segments corresponding to the rats and quantified it as much as possible. This study aimed to reduce the impact of differences in rat activity levels on experimental results.

In this study, we developed an LFH rat model in a shorter time, which effectively simulated the pathological process of LFH in humans. Comprehensive tests, including MRI, IHC, and WB, confirmed the reliability of the model. However, this study has some limitations. Future research should focus on optimizing sample processing and conducting additional independent experiments to further validate the results. Despite these limitations, the reliability of this model was demonstrated through various detection methods.

## Conclusion

A relatively reliable, efficient, and convenient rat model of LFH was established through an innovative surgical intervention combined with stress stimulation.

## Data Availability

The dataset associated with this study has been uploaded to Zenodo and is publicly accessible. The DOI for the dataset is doi: https://www.doi.org/10.5281/zenodo.14585247.
